# The spiritual needs of surgical patients in Iranian hospital settings: a qualitative study

**DOI:** 10.1186/s13104-025-07228-w

**Published:** 2025-04-10

**Authors:** Fahimeh Alsadat Hosseini, Marzieh Momennasab, Shahrzad Yektatalab, Armin Zareiyan

**Affiliations:** 1 Community Based Psychiatric Care Research Center, School of Nursing and Midwifery, Shiraz University of Medical Sciences, Shiraz, Iran; 2 Department of Nursing, School of Nursing and Midwifery, Shiraz University of Medical Sciences, Shiraz, Iran; 3 Community Based Psychiatric Care Research Center, School of Nursing and Midwifery, Shiraz University of Medical Sciences, Shiraz, Iran; 4 Public Health Nursing Department, Aja University of Medical Sciences, Tehran, Iran

**Keywords:** Qualitative study, Surgical patients, Spiritual needs, Hospitalization

## Abstract

**Background and aim:**

Surgical patients seek spiritual support because they feel threatened by their physical integrity and sense of self. Delivering spiritual care to surgical patients requires recognizing their spiritual needs. These needs can take on various forms in different cultures and religions. Thus, the present study aimed to investigate the spiritual needs of surgical patients in Iranian hospital setting.

**Method:**

This qualitative content analysis study research was conducted on 16 surgical hospitalized patients in Shiraz, Iran. The data was collected through in-depth and semi-structured interviews. The qualitative content analysis method of Graneheim and Lundman was used for analyzing the data, and Guba and Lincoln criteria were used to ensure the trustworthiness of the research data.

**Results:**

The findings of this study were presented in the form of four key categories: “Divine Resilience,” which highlights the need for a divine relationship to achieve spiritual strength; “Supportive Bonds in the Healing Process,” emphasizing the importance of interpersonal connections and support networks; “Peaceful Environment,” reflecting the necessity of a tranquil and supportive setting; and “Transcendence Beyond Difficulties,” which underscores the patients’ quest for meaning and purpose amidst their challenges.

**Conclusion:**

Surgical patients experience interconnected spiritual needs, including divine resilience, supportive interpersonal relationships, a peaceful environment, and transcendence beyond difficulties. Addressing these needs by healthcare authorities and policymakers is essential for providing holistic surgical care and enhancing patients’ overall well-being.

## Introduction

Surgical patients frequently fear for their lives or that they might lose a body part as a result of the procedure. Additionally, many fear that surgery could endanger them or make their lives difficult [1, 2]. For example, due to the change in body image in surgical patients, they feel as though their physical integrity and self-concept are in danger [3]. Additionally, patients who are having surgery for the first time frequently worry about their safety [4]. As a result of these feelings, they frequently turn to spiritual intervention during, prior to, and following surgery.

Studies have shown that spiritual intervention has a successful surgical outcome for surgical patients [5–7]. Prayer and meditation are examples of religious and spiritual practices that could be employed as non-pharmacological pain relief methods for patients recovering from Caesarean surgery and acute postoperative pain [6]. Additionally, prayer meditation promotes greater relaxation and a reduction in postoperative nausea and vomiting [6]. According to the research of Adugbire and Aziato (2020), surgical patients believed that supernatural assistance from their religious faith would be necessary for a successful surgical outcome [5]. In addition, spiritual care or intervention for the patients can lead to a reduction in stress, anxiety, and depression, as well as an increase in resilience and hope among patients [8, 9]. Therefore, it seems providing spiritual intervention or care should be a part of the health care provided to surgical patients.

Spiritual intervention and care depend on studying and meeting spiritual needs [10]. On the other hand, spiritual needs assessment is an essential part of studying holistic health care [11]. Therefore, assessing the spiritual needs is a prerequisite for spiritual care, and providing holistic care can lead to the surgical patients’ well-being.

Although nurses are responsible for patient care and have to respond to the spiritual needs of patients [12], the available documents state that the spiritual needs of outpatients and hospitalized patients are not properly met [13, 14]. If patients do not consider their spiritual needs, they may experience spiritual suffering [15]. Unmet spiritual needs are associated with a reduction in care quality, patient satisfaction, and quality of life [16]. Since surgical patients face unique challenges, such as fear of mortality, body image changes, and postoperative distress, their psychological and spiritual suffering can intensify [17, 18]. Failure to identify and address their spiritual needs can worsen this distress, delay recovery, and negatively impact their overall well-being [19]. Therefore, understanding and meeting the spiritual needs of surgical patients is crucial for providing comprehensive care and improving patient outcomes.

Many qualitative studies on the spiritual needs of patients have primarily focused on cancer patients [12, 16], those with chronic diseases [14, 15], patients at the final stage of life [20], or older adults [21]. Several qualitative studies in Iran have also investigated the spiritual needs of cancer patients [22–25] and hospitalized patients in general [25, 26]. However, there is a notable gap in research specifically assessing the spiritual needs of surgical patients within hospital settings. Based on the qualitative studies, before surgery, anxieties related to the procedure, potential complications, and uncertain outcomes often surface, prompting patients to seek meaning and strength in their faith or belief systems [27, 28]. During hospitalization, the experience of vulnerability, dependence, and physical discomfort can intensify the need for spiritual support, prompting patients to rely on prayer, meditation, or connections with loved ones and religious leaders [28, 29]. After surgery, patients may experience a range of emotions, from relief and gratitude to fear, anxiety, or even spiritual distress [28, 30, 31]. Therefore, the experience is not universally positive. Some patients experience spiritual distress, characterized by feelings of isolation, despair, and questioning of their faith or beliefs [30]. Furthermore, Puchalski’s Spiritual Care Framework emphasizes the importance of addressing spiritual needs to improve patient outcomes [32]. Given the unique health challenges faced by surgical patients [17, 18] and the potential impact of spiritual needs assessment on improving their health outcomes [5–7], addressing this gap is crucial to enhancing the quality of care and patient recovery.

Qualitative content analysis is a meaningful method to provide broad, in-depth insights about a phenomenon, its etiology, and its manifestations based on the perspective and experience of those involved [33]. Therefore, it seems that using this approach can be so helpful in exploring the spiritual needs of surgical patients. In addition, although some dimensions of spiritual needs are universal, others are culture-specific [34]. So, it is important to understand the concept of the spiritual needs of surgical patients in the context of specific cultural parameters and populations. Accordingly, the present study aimed at exploring the spiritual needs of surgical patients in hospital settings in Iran through a qualitative study.

## Materials and methods

The present qualitative study was conducted from October 2021 to May 2022. In this qualitative study, the participants were selected from three surgical wards in three private and public hospitals (the Medical Education Center affiliated with Shiraz University of Medical Sciences) in Shiraz, Iran.

The inclusion criteria were hospitalization as a surgical patient, speaking and understanding the Persian language, the age range of 18–60 years, being conscious, coherent, and oriented to time, person, and place, and having the ability to present enough and rich descriptions of the studied subjects. Exclusion criteria were AIDS or cancer, being in the final stages of life, or recent acute physical or mental disorders. The purposeful sampling method was used, and the subjects with rich and thick descriptions were used to obtain attitudes and discover a new meaning for the studied phenomenon [35]. Furthermore, sampling was conducted with the maximum variance in terms of surgery type, age, gender, and marital status.

Data collection was carried out using the personal interview method. For this purpose, 16 in-depth and semi-structured interviews were conducted with 16 patients face-to-face in a private environment (the conference hall of the ward).

To gather data, the first researcher visited a number of hospital wards, including surgical, urology, orthopaedic, and obstetrics and gynecology, on different days and across different shifts. After obtaining approval from the supervisors and head nurses, the initial researcher asked the patients who met the inclusion criteria and were prepared to participate in the study. To assess the inclusion criteria, the research team used a combination of methods. Medical records were reviewed to confirm the surgical status and age range of patients. During the initial screening and consent process, brief assessments were conducted through direct interaction, including simple orientation questions to ensure participants were conscious, coherent, and oriented. The ability to provide rich descriptions was evaluated subjectively based on the depth and clarity of responses to open-ended questions during the interviews. Professional judgment and experience were employed to determine if participants met the criteria for providing sufficient and rich descriptions. This subjective assessment was supplemented by reviewing interview content to ensure comprehensive articulation of experiences.

Each interview was unstructured at the beginning and started with general and open-ended questions like, “How have your medical condition and surgery impacted your feelings, requirements, or behaviors?” and continued with, “Since your hospitalization, what things do you think you need more?” “During hospitalization, what helps you to be happy?” Following the patient’s response, more questions were asked to elucidate the situation further. Furthermore, probing questions like “Can you describe more to help me understand you better?” and “Could you give me an example?” Depending on the responses, questions such as “What do you mean?” were also asked.

The approximate time of the interviews was about 45–60 min, and they were recorded with the permission of the patients. Immediately after the interview, the researcher listened to the recordings several times and then transcribed them. The data were then analyzed, and the next interview was planned. The interviews were continued until data saturation. In this study, data saturation was achieved when no new codes, subcategories, or categories emerged from the analysis. To ensure this, after reaching a point where successive interviews did not generate novel insights, two additional interviews were conducted to confirm that no further conceptual developments occurred. This approach aligns with qualitative research best practices, ensuring comprehensive data collection and thematic consistency. Continuous comparative analysis and iterative coding further validated saturation, strengthening the trustworthiness of the findings [36, 37].

Conventional content analysis introduced by Graneheim and Lundman was used for data analysis [38]. The main stages of content analysis were specified through selecting the unit of analysis, data organization based on open coding, classification based on the available differences and similarities, data reduction, and theme extraction [39, 40]. In this study, an open content analysis method was employed, allowing categories and subcategories to emerge directly from the data without the use of predefined codes or theoretical frameworks.

To ensure the validity and trustworthiness of the interpretation and analysis of the results, several procedures were rigorously employed throughout the study. Guba and Lincoln’s criteria [41] for trustworthiness, including credibility, transferability, dependability, and confirmability, were systematically applied. Specifically, member checking was used to review and verify preliminary findings with a subset of participants, ensuring accuracy and resonance with their experiences. Additionally, two independent researchers conducted coding and thematic analysis to enhance inter-rater reliability and minimize personal bias, with discrepancies resolved through consensus. The research team engaged in peer debriefing sessions to gain external perspectives on the data interpretation, while thick descriptions and detailed reporting of the analysis process were maintained to facilitate transferability and replication of the study. Further, the prolonged engagement approach, negative case analysis, and maximum diversity in sampling were utilized to bolster the trustworthiness of the data. A team approach was also employed to ensure that the categories accurately reflected participants’ thoughts, involving collaborative discussions and consensus-building throughout the analysis process.

### Ethical considerations

After receiving approval from the Ethics and Research Committee of Shiraz University of Medical Sciences (No. IR.SUMS.REC.1395.S872), the participants who met the study criteria were identified. After presenting oral and written explanations of the research objectives, informed consent was obtained from the subjects. Participants were offered an opportunity to share their personal experiences, contributing to a deeper understanding of the spiritual needs of surgical hospitalized patients, which could lead to improved practices and interventions. However, potential emotional discomfort from discussing personal experiences was mitigated by offering the option to withdraw at any time and providing access to counseling if needed. Confidentiality was strictly upheld through data anonymization, ensuring that participant identities were protected throughout the study. As a result, participants’ identities were not disclosed, and no identifying information was provided in the research outputs.

## Results

The interviews were conducted with 16 surgical patients in different settings in private and public educational hospitals. Most of the patients interviewed were female (62.5%) and married (68.8%). The mean and standard deviation of age were 39.50 ± 13.27 years. The personal and social information of the participants is shown in Table 1.

**Table 1:** The personal and social information of the participants

Participant	Gender	Age (year)	Marital status	Educational level	Job	Duration of hospitalization
P1	Female	28	Married	MSc	Self-employed	8 days
P2	Female	52	Married	Primary school	Housewife	5 days
P3	Female	59	Married	Primary school	Housewife	4 days
P4	Female	50	Married	Primary school	Housewife	3 days
P5	Female	42	Married	Diploma	Housewife	5 days
P6	Female	19	Single	High school	housewife	7 days
P7	Male	30	Married	Diploma	Self-employed	12 days
P8	Male	55	Single	MSc	Governmental	16 days
P9	Male	58	Married	Diploma	Retired	20 days
P10	Male	44	Single	Primary school	Self-employed	12 days
P11	Female	32	Married	Diploma	Housewife	5 days
P12	Female	50	Married	Primary school	Housewife	6 days
P13	Male	28	Single	Bachelor’s degree	Self-employed	3 days
P14	Female	24	Married	Bachelor’s degree	Housewife	6 days
P15	Male	26	Single	MSc	Engineer	12 days
P16	Female	35	Married	Diploma	Housewife	6 days

The findings were organized in the form of four main categories, including “Divine Resilience”, “Supportive Bonds in the Healing Process”, “Peaceful Environment”, and “Transcendence Beyond Difficulties” and 10 subcategories. Table 2 shows the main categories and subcategories.

**Table 2:** The categories and subcategories extracted from spiritual needs of patients

Category	Subcategory
Divine resilience	Spiritual resilience through direct relationship with God Spiritual resilience through an indirect relationship with God
Supportive bonds in the healing process	Perceiving presence Mutual support Keeping dignity
Peaceful environment	Pleasant hospital environment Natural environment
Transcendence beyond difficulties	Promoting self-integrity Searching for meaning and purpose Submission and satisfaction

### Divine resilience

The interviewees in this study needed divine relationships to gain internal peace while encountering the difficulties of hospitalization and surgery. This category includes two subcategories: “Spiritual resilience through direct relationship with God” and “Spiritual resilience through an indirect relationship with God”.

#### Spiritual resilience through direct relationship with god

The participants felt the presence of God more than ever and established a relationship with Him. They had different ways of establishing a direct relationship with God.

- *Praying calms me down. (P.4)*
- *I believe that each operation*,* even a small one*,* may cause death or some serious problems*,* so I need to pray before the operation and ask God to help me pass the operation successfully. (P.1)*
- *When someone reads the Quran to me*,* I get calm. (P.1)*
- *Our prayer is not only praising God. If I raise my hands and thank God for His blessings*,* it will work on me. (P.7)*

They considered the need for prayer to recover from disease, asked God to relieve them of pain and disease, and trusted in God to heal their disease.

- *In my opinion*,* God helps us in the first place*,* and then His tools are the doctors. I always trust in God and ask Him to help me get well soon. (P.4)*

#### Spiritual resilience through an indirect relationship with god

The interviewees also needed an indirect relationship with God. They sought recourse from their illness from the prophets and imams to get well and be relieved of the problems related to the disease and surgery.

- *When my heart is broken or I feel disabled*,* I ask our imams for more help. It causes me to feel empowered. (P.12)*

Furthermore, the surgical patients in many cases asked others to pray for them.

- *The blessing of other people is essential for improving my health and helping me tolerate the various challenges related to surgery and hospitalization; it is extremely important. (P.3)*

### Supportive bonds in the healing process

The interviewees felt the need to feel the presence and attention of others, receive others’ support, and provide support for others. Also, the patients wanted the people in the hospital setting to be treated with dignity. This main category includes three subcategories: “perceiving presence”, “mutual support”, and “keeping dignity”.

#### Perceiving presence

The participants considered the presence, attention, and interaction with the healthcare team and family as a relaxing factor, helping them to forget their problems, fears, loneliness, homesickness, and discomfort and cope with the difficult conditions of hospitalization and surgery.

- *Whenever my family and relatives pay attention to me*,* visit*,* talk*,* and laugh with me*,* it makes me happy… when they make me hopeful and happy*,* I am sure that my surgery outcomes and treatment process will be done better and with more comfort. (P.14)*
- *They are really good nurses because when I have a question about my surgery or treatments*,* they answer me in a self-contained way*,* and when I need something*,* they really satisfy me*,* which lifts my spirit. (P.6)*

In addition, the participants in this study considered the interaction with other patients necessary to improving their spirits, helping them cope with difficult situations and stress, and increasing their awareness regarding the disease and surgery.

- *When I talk to patients with the same problem or surgery*,* they give me information*,* and I see that a patient in my condition gets well easily and continues his life. Well*,* it makes me hopeful and calm*,* and I can better continue my life. (P.7).*

#### Mutual support

The participants need empathy, love, kindness, and care from the healthcare team, family, and other patients or their companions due to the disabling conditions of the disease and surgery in both physical and spiritual aspects.

- *It calms me down when the treatment team reminds me of the more difficult situations and empathizes with me… I feel happy and relaxed when I talk to my family or relatives about my worries and fears. (P.8)*
- *I needed the healthcare team to give me clear explanations about the operation and answer my concerns properly. (P. 6)*
- *This woman (another patient companion) is really like a compassionate mother who comes and feeds me. When I am thirsty*,* she gives me water and helps me as much as possible*,* and when I cry*,* she sympathizes with me. It is a very good feeling; she looks like my mother. (P.16)*

In addition, they deeply felt an internal need to support others, help other patients, sympathize with them, and sacrifice themselves for their needs.

- *Whenever I help other patients*,* I am happier and more relaxed. (P.11)*
- *When I see someone who is really in pain*,* I pray for him…. Sometimes I talk to him to pacify him and calm him down. (P.1)*

#### Keeping dignity

The participants needed a respectful hospital atmosphere that focused on maintaining the dignity of themselves and their companions, as well as that of other patients, their companions, and the treatment team. They believed that maintaining a respectful hospital atmosphere could have a significant effect on the effective interactions of patients and their recovery.

- *Everybody*,* whether the patient*,* healthcare staff*,* or others*,* certainly wants others to have respectful and good behavior*,* not a behavior that negatively affects them. (P.11)*

The participants felt the need to keep their physical privacy in front of the opposite sex, such that they felt uncomfortable when they had no appropriate clothes in the presence of the opposite sex.

- *I like to have a female nurse because she must see my body when she is dressing up my wound*,* which will be difficult for me… I always emphasize with the nurses dressing me up to close all the curtains around me because the male staff of the hospital or the male patient companions are always out there. (P.3)*

### Peaceful environment

The patients in this study needed a peaceful and pleasant environment in terms of facilities and appearance. In addition, they considered using the natural hospital environment for keeping peace and improving their spirit.

#### Pleasant hospital environment

Clean rooms with beautiful views, updated facilities, and appropriate arrangements, along with spiritual facilities like religious facilities, music, and books, made it easier for patients to tolerate the hospital environment.

- *I wish I had a larger and more beautiful room… well… a bigger and cleaner room will affect my spirit more positively…. If a patient wants to pray*,* he does not know what to do; it is so messy around here*,* and there is no place for praying. (P.8)*
- *If they play peaceful music*,* it will be so good…. They can build a library in the setting where patients can read books. (P.15)*

The participants in this study needed to spend their time in a quiet environment to improve their physical, spiritual, and mental conditions.

- *To rest and think*,* I need a quiet environment where I can be recharged mentally*,* make plans*,* and make myself hopeful. (P.8)*

#### Need for nature

Going in and watching the natural environment of the hospital and breathing the fresh air can change the spirit of the participants, increase their energy, and give them hope and happiness.

- *When you go outside*,* the weather is certainly different from the inside. You get to see the sky again and the flowers and trees*,* which will recharge your spirit. (P.9)*

### Transcendence beyond difficulties

Transcendence is another important spiritual need for surgical patients. Surgical patients tried to improve their self-integrity in difficult conditions. They further searched for or realized a transcendental meaning and purpose in the current condition and hospitalization. In addition, participants in this study were satisfied with the fact that their disease was the will of God.

#### Promoting self-integrity

Given the stressful nature of the surgery and hospitalization, the patients need certain coping strategies, which include hope, optimism, increasing patience and independence, and living in the present to promote internal integrity.

- *If the patient is optimistic about the surgery and future*,* there is no need for him to return to the past… what happened to us? We*,* as patients*,* must be independent*,* hopeful*,* and patient. (P.10)*

#### Searching for meaning and purpose

Searching for meaning and purpose is one of the most important needs of surgical patients, resulting in motivation to tolerate and cope with problems. When participants consider the health issue and hospitalization as useful and positive experiences, they realize that these painful events lead to spiritual transcendence in different aspects.

- *The experience of living in the hospital ward is good. It makes patients shy away from pride and find that life is too short not to be good.… when you are hospitalized for a few days for an operation*,* you learn a lot about tolerating pain and understanding that others have pain*,* too. These have been really effective for people like me. (P.1).*
- *Disease and my current situation bring me closer to god. (P.2).*

In addition, patients often searched for their purposes while being hospitalized, which had positive effects on them. Sometimes these purposes were materialistic, but sometimes they followed afterlife goals.

- *One of the important purposes of my life is to take care of my children. I make a lot of efforts to get well soon and get on with that. (P.12)*
- *I want others to remember my goodness after my death. (P.9).*

#### Submission and satisfaction

Most participants in this study, despite their problems and concerns, tried to be satisfied with the intentions of God regarding their disease. It leads to patience and compatibility while encountering problems during hospitalization and brings a sense of peace to patients.

- *I am satisfied with God’s satisfaction. God has given us the disease*,* and he knows the treatment. (P.12)*

## Discussion

Spiritual care is a critical component of holistic healthcare, emphasizing the importance of addressing the spiritual needs of patients for achieving comprehensive care. However, despite its recognized significance, there remains a substantial gap in the integration of spiritual aspects into care practices, particularly among surgical patients. Notably, a recent study revealed that only a small fraction (2.6%) of the identified quality indicators for surgical care directly addressed spiritual needs [40], underscoring the urgent need for standardized frameworks to address and integrate the spiritual needs of surgical patients into the overall care process. Therefore, the present study aimed to explore the specific spiritual needs of surgical patients within Iranian hospital settings, with the ultimate goal of contributing to the development of more inclusive and culturally relevant care practices.

Spiritual care is a crucial component of holistic healthcare, making the study of spiritual needs among surgical patients essential for effective spiritual care. A standardized set of quality indicators specifically addressing the spiritual dimensions of care for surgical patients has not yet been established. A very small percentage (2.6%) of the identified indicators specifically focus on addressing the spiritual needs of patients [42]. Therefore, this study aimed to explore the spiritual needs of surgical patients within Iranian hospital settings.

The findings of this study were organized into four main categories: “Divine Resilience”, “Supportive Bonds in the Healing Process”, “Peaceful Environment”, and “Transcendence Beyond Difficulties.” These categories collectively underscore the significant spiritual needs of surgical patients within Iranian hospital settings.

A comprehensive review of the literature reveals that while the spiritual experiences of patients undergoing surgery [30] and the positive impact of spirituality on surgical outcomes [5] have been explored, no study has specifically focused on the spiritual needs of surgical patients. Most studies in this field have concentrated on the spiritual needs of cancer patients [43–47], palliative care patients [48], or the spiritual needs of parents, caregivers, and family members of patients [43, 49–51]. Many of these studies utilized quantitative methods to investigate spiritual needs [44, 47, 52]. In Iran, several studies have also explored spiritual needs, primarily among cancer patients [24, 26, 53, 54] and COVID-19 patients [55], with some employing quantitative and descriptive methodologies [53, 54]. Notably, only one qualitative study has addressed the spiritual needs of hospitalized patients, without specifying disease types, and this study conceptualized these needs from the perspective of nurses working in various hospital departments, excluding surgical wards [25].

Regardless of differences in study populations and methodologies, a review of existing qualitative research on patients’ spiritual needs indicates that several studies have highlighted categories whose meanings align with some of the main categories or subcategories extracted in this study, such as connection with God [25, 26, 43, 56, 57], relationships with others [43, 48, 56, 57], and the search for meaning [43, 45, 48, 52] as fundamental spiritual needs. However, the categories explored in other studies often carry slightly different connotations compared to those identified in this study. These variations highlight the nuanced differences in how spiritual needs are understood and articulated across diverse contexts and populations. Moreover, in this study, additional categories such as ‘Peaceful Environment’ and ‘Transcendence Beyond Difficulties’ were identified, along with specific subcategories related to these categories, which have not been extensively emphasized in previous studies. These differences underscore the varied ways spiritual needs are conceptualized, especially in the context of surgical patients. Given the aforementioned considerations, the lack of qualitative research specifically addressing the spiritual needs of surgical patients, and the established benefits of addressing spiritual needs in improving patient health outcomes, this study contributes to the existing body of knowledge by offering new insights into the spiritual needs of surgical patients. In the following sections, the categories identified in this study will be discussed in more depth, with reference to relevant literature.

The need for **divine resilience**, or in other words, resilience through both direct and indirect divine connection, emerged as a central spiritual requirement for the patients in this study. Addressing this need was instrumental in fostering a sense of peace and patience among the patients as they faced the anxieties and threats associated with illness and surgery. This finding is consistent with prior research, which has demonstrated that Muslim patients often engage in a direct relationship with God through worship, prayer, and recitation of the Quran during hospitalization [25, 58]. Furthermore, a study by Adugbire et al. (2020) revealed that Muslim surgical patients relied on prayer to Allah before, during, and after their procedures, which helped them manage stress and maintain emotional stability [5]. Moreover, maintaining religious well-being by considering personal religiosity and one’s relationship with God enables patients to find meaning and resilience throughout the challenging medical process [59]. O’Connor (2017) also observed that when surgical patients prayed with healthcare staff before surgery, they consistently reported feelings of happiness and being cared for [60]. Other qualitative studies have found that religious involvement is closely linked to the pursuit of hope and faith, especially in the context of cardiac surgery, where spiritual resources are highly valued [61, 62]. In the current study, the methods of prayer varied among patients, from performing obligatory religious rituals to engaging in more personal, flexible forms of communication with God, such as informal conversations. These variations are in line with the findings of Aseleh Jahromi and Akbar (2020), who similarly noted a range of prayer practices among patients [25]. This diversity underscores the importance of personalized approaches in assessing and addressing the religious needs of patients.

Additionally, a significant number of patients in this study expressed a need for resilience through an indirect relationship with God, such as seeking intercession or requesting prayers from others. In Shiite Iranian culture, the concept of recourse—seeking intercession from prophets and imams before God for forgiveness and healing—holds considerable significance [63]. Many patients in this study requested prayers from others, believing in the efficacy of such prayers for their well-being. This practice aligns with other studies where family members and relatives prayed for the patient’s successful surgical outcome, reinforcing the belief in the power of intercessory prayer [1, 5]. Intercessory prayer, where a second person or group prays to God on behalf of someone in need, has been shown to have a positive impact on patients’ well-being and health [64]. Therefore, this indirect relationship with God through others’ prayers is a vital aspect of spiritual care that should not be overlooked in the context of surgical patients.

Overall, findings from this study align with previous research indicating that prayer and connection with God help reduce stress and enhance emotional stability in surgical patients. However, compared to prior studies, this research places greater emphasis on individual differences in spiritual coping, including the diversity of prayer practices and the role of intercessory prayer, aspects that have been less explored in Western studies.

The need for resilience through divine connection, whether direct or indirect, appears to be particularly pronounced during times of illness and surgery [65]. Patients in this study perceived that meeting their spiritual needs was crucial to achieving successful surgical outcomes [5]. This perception is rooted in the cultural and religious context of Iran, where religion is deeply intertwined with societal values and relationships [66]. As a result, incorporating religious elements into the care of surgical patients is imperative. However, the literature also reveals that nurses often neglect patients’ religious beliefs during preoperative preparation [5]. Addressing this gap is essential for reducing anxiety, fostering hope, and improving surgical outcomes for patients [67, 68]. Consequently, healthcare authorities must ensure that adequate support and facilities are provided to accommodate religious practices and spiritual rituals within hospital settings, particularly for surgical patients.

In this study, the category of **establishing supportive bonds during the healing process** emerged as a critical spiritual need among patients. These supportive bonds were not limited to mere physical presence; instead, they encompassed a profound connection characterized by attention, respect, and an understanding of the patient’s dignity and various needs. Patients expressed a need for meaningful connections with their relatives, the healthcare team, and even other patients. Moreover, they demonstrated a strong desire to support fellow patients as part of their humanistic responsibility.

The significance of these interpersonal connections aligns with previous research. For instance, Lormans et al. (2021) identified ‘being connected’—including connections with family, friends, the broader community, and the outside world—as one of the primary spiritual needs of patients with life-limiting illnesses [48]. Similarly, Jadidi et al. (2022) found that the need to connect with others was a key spiritual requirement among Muslim older adults. While these findings share similarities with the present study, it is important to note that these studies were conducted on non-surgical patient populations [48, 69]. Phillips-Salimi and Haase (2012) identified key attributes of connectedness in patient-provider relationships, including intimacy, sense of belonging, caring, empathy, respect, trust, and reciprocity [70]. These attributes are essential for fostering a healing environment and supporting patients’ psychosocial well-being. A substantial body of literature highlights the positive impact of family members’ presence on patients’ clinical and psychosocial outcomes [71]. Family members and other supporters, often referred to as “care partners” for hospitalized surgical patients, play a crucial role in providing non-medical care that is vital for maximizing in-hospital recovery [72–75]. The absence of care partners at the bedside creates a significant gap in the resources available to patients and their healthcare team [46]. Additionally, Hosseini et al. (2019) emphasized that the connection with and holistic presence of healthcare teams, family members, and other patients is a primary spiritual need with positive health implications for patients [76]. Similarly, Hesselink et al. (2020) highlighted the importance of interpersonal healing environments, including supportive contacts with care providers, patients, and relatives, for postoperative healing [77]. Birkelund and Larsen (2013) further supported this finding, noting that helping other patients and engaging in mutual emotional support were central to creating a supportive environment [78].

The need for connectedness, as described in various studies, underscores the interpersonal dimension of spiritual needs, which is intricately linked to other spiritual aspects [79–81]. In many Islamic teachings, the importance of presence, attention, support, and dignity is emphasized, reflecting the cultural and religious values that shape patients’ spiritual needs in the Middle East and other emerging nations [82–84]. These regions often have large extended families deeply involved in patient care, which must be considered in the spiritual needs assessment [82–84].

Finally, the critical role of connectedness in maintaining patients’ integrity and internal balance has been well documented [85]. While the findings of the present study align with previous research on the significance of interpersonal connections in spiritual care, they extend the discussion by emphasizing the unique needs of surgical patients. Unlike studies focused on non-surgical or terminally ill populations, this research highlights the role of care partners, healthcare teams, and peer support in fostering resilience and recovery in a surgical context. Given the cultural importance of family involvement in patient care, particularly in Middle Eastern settings, a comprehensive approach to spiritual support should incorporate these interpersonal dimensions. Strengthening healthcare policies to facilitate meaningful connections can enhance both the psychological and physical well-being of patients during their healing journey. Therefore, educating healthcare teams on the principles of compassionate communication and patient-centered care, ensuring the effective presence of family members, and creating an environment that nurtures meaningful relationships among patients are essential steps in addressing their interconnectedness needs during recovery.

In this study, **the need for a peaceful environment** emerged as a significant spiritual requirement for surgical patients. This finding aligns with previous research highlighting the importance of environmental factors in the healing process [86, 87]. Hesselink et al. (2020) identified that connections with the external environment, such as enjoying natural views and engaging in activities like getting fresh air, are crucial for optimal postoperative healing [77]. The comfort and aesthetics of the hospital environment—including its cleanliness, appropriate view, and privacy—contribute to improving patients’ conditions and support their healing [88].

Further supporting this, Erichsen (2013) found that immersion in the beauty of nature was one of the highest spiritual needs reported by patients, underscoring the profound impact of natural surroundings on spiritual well-being [89]. Ulrich (1984) also demonstrated that exposure to natural landscapes not only reduces the length of hospital stays but also enhances patients’ evaluations by the healthcare team, leading to less medication use and fewer side effects from surgery [90].

The findings of this study align with previous research emphasizing the role of a peaceful environment in patient recovery. However, unlike prior studies that primarily focused on general or chronically ill patients, this study underscores the unique needs of surgical patients, who may experience heightened vulnerability and distress due to the nature of their medical procedures. This distinction highlights the necessity of tailored environmental modifications in surgical wards to foster a healing atmosphere and support both physical and spiritual well-being. Additionally, a peaceful environment may contribute to fulfilling other spiritual needs, such as enhancing self-integrity. Thus, creating and maintaining a peaceful environment is not merely a comfort but a fundamental aspect of addressing the spiritual needs of surgical patients. Therefore, it is crucial for healthcare settings to prioritize the establishment of a serene and aesthetically pleasing environment to support the holistic well-being and recovery of patients.

Another category identified in the spiritual needs of patients in this study was **transcendence beyond difficulties**. This concept aligns with findings from Hanley and Garland (2022), who suggest that transcendence, often achieved through mindfulness-based therapy, can significantly enhance clinical outcomes for a broad range of surgical patients [91]. In this study, participants employed various coping strategies to strengthen their spiritual well-being and maintain internal integrity. These strategies are consistent with other research indicating that spiritual needs such as forgiveness [92], hope [93], and living in the present moment [94] are crucial for coping with health challenges.

Moreover, many participants found spiritual meaning in their illness and surgery, viewing these experiences as opportunities to derive greater purpose and resilience. This aligns with previous studies highlighting the search for and understanding of meaning throughout the illness trajectory as a fundamental spiritual need among hospitalized patients [43, 45, 48, 52]. However, as these studies primarily focused on non-surgical patients and given the limited research specifically on surgical patients, findings from these studies with other patient populations have also been referenced. Guerrero-Torrelles et al. (2017) highlighted that finding meaning and purpose is a prominent spiritual aspect that helps individuals cope with disease [95]. Physical illness often acts as a catalyst for introspection, prompting individuals to address existential questions and reassess priorities across physical, psychological, social, and spiritual dimensions [96].

Cultural and religious backgrounds also play a significant role in how patients achieve transcendence. In this study, participants who believed in the supremacy of God’s will and wisdom found comfort in surrendering to divine intentions, which they considered a form of spiritual fulfillment and a path to ultimate salvation. This surrender and satisfaction are seen as high levels of spiritual achievement [97], reflecting a deep sense of spiritual contentment and purpose.

The category “Transcendence Beyond Difficulties” aligns with previous research on spirituality in coping with illness. However, unlike some relevant studies on cancer, chronic or palliative care patients [48, 52, 95, 96], our findings highlight surgery as a catalyst for spiritual transformation. While prior research emphasizes secular perspectives, this study underscores the unique role of cultural and religious beliefs, particularly surrendering to divine will, in achieving transcendence. Moreover, while strategies like forgiveness, hope, and mindfulness [92–94] are well-documented, our findings suggest they hold distinct significance in the surgical context, emphasizing faith and resilience. These insights highlight the need for further research on spiritual transcendence in diverse clinical settings.

### Limitations

Despite introducing diversity in the sample, with participants recruited from both private and public hospitals, which was intended to enhance the comprehensiveness of the qualitative data, the varying facilities and services available in these hospitals may influence the spiritual needs of patients. This variability represents a limitation of the study. Future research could focus on specific hospital types or explore more homogenous patient populations to gain a deeper understanding of the impact of hospital environment on patients’ spiritual needs. Additionally, incorporating alternative data collection methods, such as direct observation and interviews with healthcare professionals and family members, could provide a more comprehensive and nuanced perspective on the spiritual needs of hospitalized patients.

## Conclusion

The findings of this study were organized into four key categories: “Divine Resilience,” “Supportive Bonds in the Healing Process,” “Peaceful Environment,” and “Transcendence Beyond Difficulties.” These categories reveal that the spiritual needs of surgical patients encompass transcendental, interpersonal, intrapersonal, and environmental dimensions, which are interrelated and cannot be addressed in isolation. Thus, a comprehensive approach to spiritual care is essential for effectively meeting these needs.

This study highlights that while some spiritual needs are universal, others are profoundly shaped by cultural and religious contexts. Even within similar overarching categories, the specific ways in which patients experience and express their spiritual needs can differ significantly based on these contextual factors. For instance, the category of “Divine Resilience” in this study extends beyond general notions of psychological strength and coping; it is deeply intertwined with the concept of surrendering to divine will, perceiving suffering as a test of faith, and finding meaning through religious devotion—nuances that may not be as prominently emphasized in studies conducted in secular or culturally distinct settings. These findings underscore the importance of considering sociocultural and religious backgrounds when addressing the spiritual needs of surgical patients and highlight the need for further research in diverse cultural and clinical contexts. Health officials and policymakers can leverage these findings to address the multifaceted spiritual needs of surgical patients in various hospital settings, ultimately advancing holistic care and improving patient well-being.

## Data Availability

No datasets were generated or analysed during the current study.
